# Multifunctional phototheranostic agent ZnO@Ag for anti-infection through photothermal/photodynamic therapy

**DOI:** 10.3389/fchem.2022.1054739

**Published:** 2022-11-09

**Authors:** Enoch Obeng, Jiayao Feng, Danyan Wang, Dongyang Zheng, Bailin Xiang, Jianliang Shen

**Affiliations:** ^1^ School of Ophthalmology and Optometry, School of Biomedical Engineering, Wenzhou Medical University, Wenzhou, Zhejiang, China; ^2^ Ningbo Eye Hospital, Ningbo, Zhejiang, China; ^3^ Wenzhou Institute, University of Chinese Academy of Sciences, Wenzhou, China; ^4^ College of Chemistry and Materials Engineering, Huaihua University, Huaihua, China

**Keywords:** ZnO@Ag nanocomposite, photothermal therapy, photodynamic therapy, synergistic effect, anti-infective therapy

## Abstract

To overcome the limitations of traditional therapeutics, nanotechnology offers a synergistic therapeutic approach for the treatment of bacterial infection and biofilms that has attracted attention. Herein, we report on a ZnO@Ag nanocomposite with good biocompatibility synthesized by doping ZnO NPs with silver nanoparticles (Ag NPs). ZnO@Ag nanocomposites were synthesized with varying ratios of Ag NPs (0.5%, 2%, 8%). Under the same experimental conditions, ZnO@8%Ag exhibited outstanding properties compared to the other nanocomposites and the pristine ZnO NPs. ZnO@8%Ag demonstrated excellent photothermal and photodynamic properties. Also, ZnO@8%Ag demonstrated over 99% inhibition of *Staphylococcus aureus* (*S. aureus*) under photothermal therapy (PTT) or photodynamics therapy (PDT) as a result of the excessive generation of reactive oxygen species (ROS) by the Ag^+^ released, while the pristine ZnO showed an insignificant inhibition rate compared to the PBS group (control). Furthermore, ZnO@8%Ag completely disrupted *S. aureus* biofilm under a combined PTT/PDT treatment, a synergetic trimodal therapy, although the molecular mechanism of biofilm inhibition remains unclear. Hence, the excellent photothermal, photodynamic, biocompatibility, and bactericidal properties of ZnO@8%Ag present it as an appropriate platform for bacterial and biofilm treatment or other biomedically related applications.

## 1 Introduction

Bacterial infection is a principal problem of great concern to public health as a result of the inability of traditional antibacterial therapies to completely or efficiently eliminate bacterial infections that form biofilm over time. The approach was a therapeutic practice in the 1930s (Boucher et al., 2009; Courtney et al., 2016; Jia et al., 2017), but its current ineffectiveness has been attributed to environmental pollution and the uncontrolled use of antibiotics that have contributed to the resistance mounted by bacteria. As a result, a global crisis is facing the world with billions of dollars of estimated annual costs (Stewart and Costerton, 2001; Darouiche, 2004; Wright, 2011). Hence, researchers are responding to an urgent call for an alternative therapy to address the shortcomings of the traditional antibacterial approach. The nanotechnology-based antibacterial approach has presented a novel strategy for eradicating bacteria and, in so doing, attracted much attention, owing to its antibacterial activity and stability (Pelgrift and Friedman, 2013; Duncan et al., 2015; Liu et al., 2016). Their material presents unique characteristics such as adjustable size, shape, and structure that offer an antibacterial advantage. Not all nanomaterials have the intrinsic ability to kill bacteria. However, silver nanoparticles (Ag NPs) bear this ability and are capable of influencing cell membrane integrity, respiration, and the ATP production of the bacteria due to their shape, size, and surface charge (Morones et al., 2005; Vikesland and Wigginton, 2010; Hassan et al., 2012; Rajavel et al., 2014; Tian et al., 2014). Ag NPs exhibit tremendous effectiveness against a wide variety of microbes that are Gram-positive and Gram-negative inclusive through the release of silver ions (Ag^+^). This results in the generation of reactive oxygen species (ROS) and, at the same time, facilitates wound healing through the induction of metalloproteinase activity and enhanced neutrophil apoptosis (Kirsner et al., 2001; Chaloupka et al., 2010; Chernousova and Epple, 2013; Zheng et al., 2016; Sarfraz and Qayyum, 2018).

Zinc oxide nanoparticles (ZnO NPs) have acquired great attention and have extensively been studied due to their physical, chemical, high reflective index, and easy means of synthesis. However, their wide bandgap (3.37 eV), large exciton binding energy (around 60 MeV), low charge separation efficiency, and fast recombination rate present limitations, making ZnO NPs an inactive photocatalyst within a visible light spectrum but active in the UV region (Huang et al., 2001; Kudo and Miseki, 2009; Grumezescu et al., 2014; Ali et al., 2019). Intriguingly, modification of the ZnO NPs with noble metals such as Au, Ag, Ru, and Pt helps circumvent these drawbacks (Zheng et al., 2008; Yuan et al., 2010; Zhang et al., 2010). Studies indicate that modification of the ZnO NPs with noble metals allows the formation of a Schottky barrier to promote photogenerated charge separation, subsequently improving the material’s photocatalytic activity and making it an appropriate choice. Although UV may be required for ROS generation, the insufficient penetration depth and possible tissue damage calls off UV light for an alternative approach to a photo-responsive approach for antibacterial therapy (Kuriakose et al., 2014; Lu et al., 2016; Sun et al., 2019).

Photothermal therapy (PTT) and photodynamic therapy (PDT) are the photo-responsive bacteria-killing methods that have been used in recent years. The near-infrared (NIR) range from 700 to 1000 nm (biological window) may present a controllable cause of tissue damage and an efficient therapeutic effect, depending on their mode of usage (Chang et al., 2013; Hou et al., 2015; Li et al., 2018). However, the single-modal use therapy of the former has been reported to pose a high risk of thermal damage or inflammation (Yang et al., 2017; Zhang et al., 2018; Liu et al., 2019a; Gao et al., 2019), making the PTT-based multimodal therapy with NP dosage the best alternative due to reduced irradiation time and improved antibacterial efficiency. In such instances, the enhanced ROS generation contributes to the multimodal therapy approach for bacteria eradication (Hou et al., 2012; Liu et al., 2019b; Wang et al., 2019). The synergism of the PTT and PDT also presents a great advantage and is considered a trimodal therapy.

In this study, we fabricated ZnO NPs and ZnO@Ag nanocomposite doped with different molar ratios of Ag (0.5%, 2%, 8%), using silver nitrate (AgNO_3_) as the precursor for improving the charge transfer and ROS generation. For the first time, we report on the bacterial inhibition response. ZnO@8%Ag was later selected due to its excellent photo-responsiveness under NIR irradiation and biocompatibility. The previous research report indicates that the best photocatalytic activity of Ag in ZnO@Ag was evident when the Ag content was 8% (Nie et al., 2020). ZnO@8%Ag’s excellent performance under PTT and PDT together with the biocompatibility performance was investigated. Also, the synergistic effect of ZnO@8%Ag in combination with PTT and PDT against *Staphylococcus aureus* (*S. aureus*) *in vitro* and *in vivo* was investigated. *In vitro* experiments revealed that, under NIR irradiation (808 nm), ZnO@8%Ag exhibited a tremendous bactericidal effect against *S. aureus*. The effect was similar under PDT treatment of ZnO@8%Ag. Both displayed a bacterial inhibition of over 99%, revealing the synergistic effect for rapid and improved release of Ag^+^. However, the impact of each multimodal treatment of ZnO@8%Ag was the same even after the combination of both PTT and PDT in a trimodal approach. Intriguingly, the biofilm test proved ZnO@8%Ag could disrupt a junk portion of *S. aureus* biofilm even at a low concentration but revealed the complete disruption of biofilm under PTT + PDT trimodal treatment. This finding revealed that a prolonged time is required to release Ag^+^ upon irradiation. *In vivo* examination confirmed that ZnO@8%Ag could effectively compromise *S. aureus* in an infected wound while accelerating the wound healing. In all, ZnO@8%Ag’s outstanding biocompatibility and synergistic effect in combination with PTT and PDT for the release of Ag^+^ and wound healing demonstrate an efficient and potential platform for the treatment of bacteria-infected wounds.

## 2 Experimental section

### 2.1 Materials and reagent

Zinc nitrate (Zn(NO_3_)_2_ 6H_2_0) >99.0 purity, silver nitrate (AgNO_3_) > 99% purity, sodium hydroxide (NaOH) > 99%, and citric acid (C_6_H_8_O_7_) >99.0 purity were purchased from Sigma-Aldrich. All other chemicals and reagents were purchased from Aladdin Chemical Reagent Co., Ltd. (Shanghai, China). All working solutions were used in their originally received state without further modification. Other preparations were made with deionized water.

### 2.2 Synthesis of ZnO and ZnO@Ag (0.5%, 2%, and 8%)

ZnO was prepared as reported by Bai et al. (2017) and Nie et al. (2020). During the synthesis process, citric acid was used as fuel. Zinc nitrate and silver nitrate served as the oxidizing reactant, and sodium hydroxide was used to adjust the pH between 12 and 13. A stoichiometric ratio of oxidizers (zinc nitrate) to the citric acid was added to a petri dish and mixed with 40 ml H_2_O. The mixture was stirred for a few minutes to develop uniformity. The dish containing the mixture was introduced into a muffled furnace maintained at 400°C. The mixture was made to undergo dehydration and decomposition under the smoldering combustion reaction to give a nanocrystalline ZnO within 5–7 min. The product was further calcined at 500°C for 3 h. A similar procedure was used in the synthesis of the ZnO@Ag with the mole ratio of 1 ZnO to 0.005, 0.02, and 0.08 of Ag to give ZnO@0.5%Ag, Zno@2%Ag, and ZnO@8%Ag.

### 2.3 Characterization

The morphology of the samples was characterized with a Tecnai G2 F20 microscope to obtain the high-resolution transmission electron microscopy (HRTEM) images. The X-ray diffraction patterns (XRD) were determined with a D/MAX 2005 Rigaku X-ray diffractometer (Japan). The XPS was determined with a Thermo Fisher X-ray photoelectron spectrophotometer K-alpha (United States), and the UV-Vis spectroscopy was performed with a CARY 5000 spectrophotometer (Agilent Technologies, United States).

### 2.4 Photothermal and photodynamic properties

Detection of ^1^O_2_ and the peroxidase-like activity after PTT treatment was conducted using 1,3-diphenylisobenzofuran (DPBF) and *o*-phenylenediamine (OPD) as chemical probes. The steady-state kinetic assay for PBS, ZnO, and ZnO@8%Ag with/without PTT treatment was determined after coincubation. A measure of 10 µL (100 μg/ml) of DPBF and OPD were separately added to the samples and subsequently analyzed with the UV-Vis spectrophotometer. The absorbances of DPBF and OPD were collected at 410 and 492 nm, respectively. The results were analyzed and plotted with GraphPad Prism 8.0.

### 2.5 Photothermal performance and photothermal conversion efficiency

A gradient concentration of the ZnO@8%Ag aqueous solution (100 µL) in a 0.5-ml Eppendorf tube was irradiated with NIR laser light (808 nm) at power densities of 0.5, 1.0, and 2.0 W/cm^2^. The temperature of the solution was monitored and recorded with a thermal imager. The temperature of the PBS and ZnO were also monitored and recorded using the same approach following the irradiation of the different laser power densities used (0.5, 1.0, and 2.0 W/cm^2^). The turn-off/cooling cycle for ZnO@8%Ag (100 μg/ml) using a 2.0 W/cm^2^ laser was accomplished with an irradiation time of 10 min, repeated five times. The photothermal conversion efficiency of ZnO@8%Ag was determined as follows.

$$\eta =hs\left(T_{\max}-T_{0}\right)/\left(I\left(1-10^{-A\lambda }\right)\right)$$

where η represents the photothermal conversion co-efficient; A is the surface area of the container; $T_{\max}$ is the maximum steady temperature, $T_{0}$ is the minimum steady temperature; I is the laser power, and $A_{\lambda }$ is the value of absorbance of ZnO@8%Ag at 808 nm.

$$\tau_{s}=\sum mC_{p}/hA\;meanwhile\;hA=\sum mC_{p}/\tau_{s}$$

where $\tau_{s}$ represents the slope of the fitting line between t and −Inθ , m is the mass of ZnO@8%Ag, and $C_{p}$ is the specific heat capacity.

$$\theta =T-T_{sur}/T_{\max}-T_{0}$$

T represents the corresponding cooling time temperature.

### 2.6 Cell culture and cytotoxicity

Murine fibroblast cells (L929) purchased from America Type Culture Collection (ATCC) were used to evaluate the cytotoxicity of the nanocomposite. Cells were cultured with Dulbecco modified Eagle medium (DEME) at 5% CO_2_ and 37°C with 10% fetal bovine serum (FBS), penicillin (100 units m/L), and streptomycin (100 mg/ml). L929 cells at a density of 5 × 10^3^/well were seeded into a 96-well plate and cultured for 24 h. Different concentrations of ZnO@8%Ag (0, 50, 100, and 200 μg/ml) were suspended in DEME, added to the 96-well plate, and irradiated with 2 W/cm^2^ lasers for 5 min. Following 48 h of incubation, 10 µL of MTT solution (5 mg/ml) was added to the solution, and PBS was used as a control. A microplate (Varioskan LUX multimode microplate reader, Thermo Fisher Scientific Co., United States) was used to measure the absorbance at 570 nm after 4 h of incubation. The viability of the control cells was estimated at 100% to determine the relative viability of the other cells.

$$Cell\;Viability\left(\%\right)=\frac{A_{e}}{A_{o}}\times 100\%,$$

where $A_{e}$ and $A_{o}$ denote the experimental and the control groups, respectively.

### 2.7 *In vitro* antibacterial test

*S. aureus* (New man strain) was used as a model strain for the evaluation of the antibacterial properties of the nanomaterials. *S. aureus* was grown in a sterilized tryptic soy broth (TSB) and solid broth (SB). A mixture of bacteria concentration of 50 µL define by the O.D_600_ = 0.05 with 50 µL of ZnO, ZnO@8%Ag, and PBS were separately added to a 96-well plate. Following 30 min of incubation at 37°C, the mixtures in the plate were irradiated with an 808 nm laser (2.0 W/cm^2^) for 10 min and then incubated for 2 h. After serially diluting the mixtures with sterile LB, 100 µL of each sample was spread on an agar plate. Following 12 h incubation at 37°C, colonies formed were counted for further evaluation.

$$Survival\;rate\left(\%\right)=\frac{{CFU}_{e}}{{CFU}_{o}}\times 100\%,$$

where ${CFU}_{e}$ and ${CFU}_{o}$ represent the number of colonies formed in the experimental and control groups, respectively.

#### 2.7.1 Bacteria morphology study

The morphologies of the bacteria treated with PBS and the nanomaterials (ZnO and ZnO@8%Ag) with/without PTT were examined with SEM. These varying solutions of bacteria were fixed with 2% glutaraldehyde for 4 h and subsequently dehydrated with a series of ethanol solutions (50%, 70%, 90%, 95%, and 100%), 10 min for each dehydration step. Samples were blown dried under nitrogen gas; silicon wafers were coated with ultrathin gold by sputtering, and the morphology of the bacteria was imaged at 3.0 kV with a Hitachi Su 8010 instrument, Japan.

#### 2.7.2 *In vitro* antibacterial evaluation with confocal microscopy

Bacterial suspension treated with different materials was collected by washing with PBS and centrifugation. Aliquots (10 µL each) of calcein acetoxymethyl ester (calcein-Am, 10 μg/ml, Solarbio) and propidium iodide (PI, 10 μg/ml, Solarbio) were incubated with 20 µL of the collected bacterial suspension in room temperature for 15 min. Fluorescent images of bacteria samples were captured by confocal microscopy (Nikon A1 Confocal Laser Scanning Microscope, Japan) and analyzed with NIS—Element viewer, Nikon.

### 2.8 *In vitro* anti-biofilm test

*S. aureus* suspension in TSB was cultured for 48 h at 37°C in a 6-well plate overnight to form a static biofilm. Bacterial cultures were diluted with tryptic soy LB OD_600_ = 0.05 and incubated in a 96-well plate for 24 h at 37°C. Following incubation, the bacterial suspension was removed, and the well was rinsed three times with PBS. Aliquots (50 µL each) of PBS and the materials (ZnO, ZnO@8%Ag) were added to the wells and treated with/without PTT or PDT for 10 min. After the wells were rinsed three times with deionized water (ddH_2_O), crystal violet (0.05%, 50 µL) was added to each well and incubated for 15 min. The dye was discarded and unbounded by rinsing with PBS three times. Acetic acid (33%, 200 µL) was added to each well to release the dye bound to the remaining biofilm for quantification. Quantification was performed with a Thermo Fisher Scientific Multiskan Sky microplate reader, OD_550_. Each experiment was performed in triplicate.

#### 2.8.1 Anti-biofilm evaluation with confocal microscopy

A coverslip was dropped at the bottom of a 24-well plate. A 20 µL aliquot of the *S. aureus* bacteria OD_600_ = 0.05 was added to the well, followed by 1000 µL of TSB, and the samples were incubated for 24 h. The coverslip was removed and rinsed with PBS three times, following treatment of the coverslip with PBS, ZnO, and ZnO@8%Ag with/without PTT/PDT. The biofilm was rinsed once again with PBS, and the coverslip was stained with 2 µM calcein-Am and 1 µM propidium for 15–20 min. Fluorescent images of bacteria were captured with a Nikon A1 confocal laser scanning microscope, and the images were analyzed with NIS—Element viewer, Nikon. Bacteria with intact and damaged membranes appeared green and red, respectively.

### 2.9 Hemolysis assay

A hemolysis assay was performed with the fresh blood of a healthy mouse by collecting blood from the eyeballs. The blood was centrifuged (12,000 rpm, 5 min) and rinsed with PBS (0.02 M, pH = 7.4) three times. The red blood cells (RBC) obtained were diluted to a 4:5 volume ratio with PBS, water, and varying concentration of ZnO@8%Ag (20, 25, 75, 100, 125, and 200 μg/ml). The PBS and water acted as the positive and negative controls, respectively. The mixtures were carefully suspended and incubated at 37°C for 1 h. Samples were centrifuged at 3000 rpm for 5 min. The supernatant was collected, and the absorbance was determined at OD_595_. The hemolysis rate was calculated according to the following equation:

$$Hemolysis\;rate\left(\%\right)==\frac{{OD}_{e}-{OD}_{-}}{{OD}_{+}-{OD}_{-}}\times 100\%$$

where ${OD}_{e}$ , ${OD}_{-}$ , and ${OD}_{+}$ represent the OD of the treatment group, the OD of the negative control group, and the OD of the positive control group, respectively.

### 2.10 *In vivo* antibacterial and wound healing

All animal experiments were performed according to the guidelines and rules approved by the Animal Ethics Committee of Wenzhou Medical University (SYXK-2021-0020).

To further evaluate the practical application of ZnO@8%Ag, BALB/c mice at 7–8 weeks were divided into five groups: PBS, ZnO, ZnO + PTT, ZnO@8%Ag, and ZnO@8%Ag + PTT. Following anesthesia, a wound (4 mm in diameter) was created at the back of the mice and injected with (20 μL, 1 × 10^8^ CFU m/L) of *S. aureus* to establish the mouse model. The surface of the wound was covered with PBS and the nanomaterials and further exposed to NIR irradiation for 10 min. This process was monitored and recorded with a thermal imager. Images of the nature and state of the wound were taken for 0, 2, 4, 6, and 10 days. ImageJ was used to monitor the different treatments for the groups.

### 2.11 Tissue toxicological analysis

A histological examination of the skin and other internal organs (lung, liver, kidneys, and heart) was conducted after 10 days. These organs were fixed with 4% paraformaldehyde; organs were sectioned and stained with hematoxylin and eosin (H&E). Samples were observed with an Olympus BX51 fluorescence imaging microscope. Fluorescence imaging was conducted with an Olympus BX51 fluorescence microscope (Olympus Co., Ltd., Japan).

### 2.12 Statistical analysis

Experimental data were expressed as mean ± standard deviation (SD). Statistical analysis among groups was evaluated with Student’s t-test, with the difference being statistically significant when *p* < 0.05 and highly significant when *p* < 0.01. The graph plots and analysis were performed using GraphPad Prism 8.0 (Graph Pad Software, United States).

## 3 Result and discussion

### 3.1 Synthesis and characterization of nanomaterials

**FIGURE 1:**
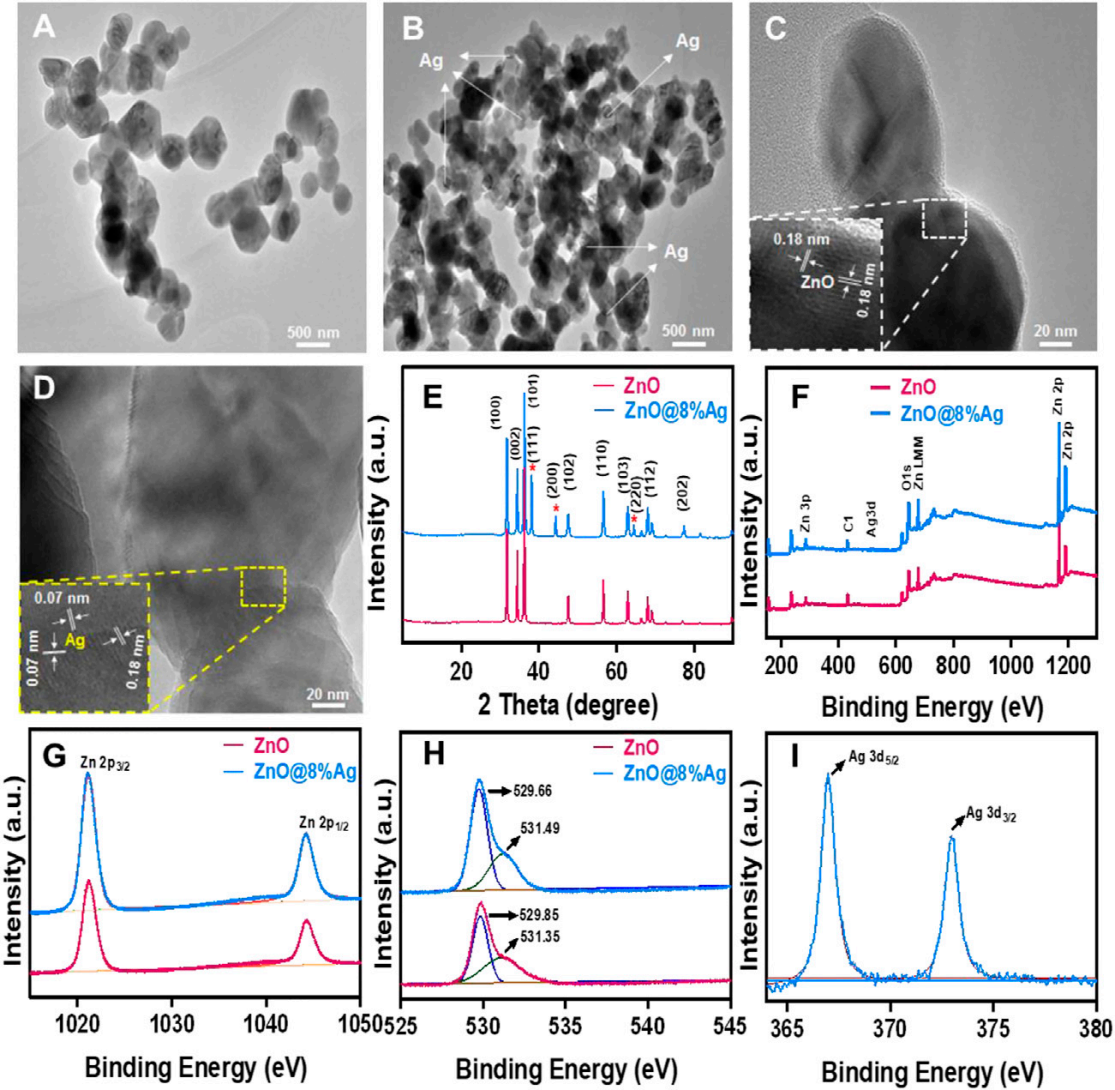
Characterization of ZnO and ZnO@8%Ag. **(A,B)** TEM images, **(C,D)** HRTEM images, **(E)** X-ray diffraction (XRD) patterns, **(F)** full XPS survey spectrum of ZnO and ZnO@8%Ag, **(G)** XPS spectra of Zn 2p region, **(H)** O1s region, **(I)** Ag 3d region.

The nanocomposites were successfully prepared, and the TEM images showed the morphology and structures of ZnO and ZnO@8%Ag. This shows that the ZnO is characterized by a size range of 74.18–161.20 nm with an average size of 109.26 nm (Figure 1A). Similarly, Figure 1B shows that the ZnO@8%Ag has an average size of 43.33 nm with a range of 31.01–55.91 nm, exhibiting the good deposition of the Ag on the surface of ZnO without agglomeration and growing in the direction that is misaligned from the normal direction. The HRTEM of ZnO shows an interplanar spacing of 0.18 and 0.07 nm in the direction of ZnO along (110) and Ag along (220), respectively (Figures 1C,D). The crystallinity, structural phase, and purity of ZnO and ZnO@8%Ag nanocomposites were identified by the XRD patterns for ZnO indexed to a hexagonal wurtzite structure (JCPDS 36-1451), where the diffraction peaks corresponding to (100), (002), (101), (102), (110), (103) (112), (201), and (202) appeared at the 2θ with values of 31.66°, 34.45°, 36.24°, 47.60°, 56.57°, 62.74°, 67.93°, 69.12°, and 77.29° (Figure 1E). In addition, the doping of ZnO unveiled distinct peaks marked with (*) in the ZnO@8%Ag at 38.23°,44.21°, and 64.34° that corresponded to (111), (200), and (220). The presence of well-defined peaks demonstrated the absence of impurities in the nanocomposites.

The appearance of new peaks in the ZnO@8%Ag nanocomposite demonstrated that the Ag^+^ with a high atomic radius had displaced the Zn^2+^, showing the successful incorporation of Ag^+^ into ZnO**.** XPS was employed to further evaluate the surface structure and chemical compositions of the nanocomposites. The full XPS spectrum shows the presence of Zn, O, Ag, and C elements, further demonstrating the successful synthesis of the nanocomposite without impurities (Figure 1F). The XPS spectrum of Zn 2p reveals binding energy peak positions of Zn2p with 1021.05 and 1044.12 eV in ZnO and 1021.13 and 1044.12 eV in ZnO@8%Ag. These peaks resemble each other, further confirming Zn in the nanocomposites (Figure 1G). This phenomenon was evident in element O, and the presence of element C could be attributed to the XPS instrument (Figure 1H). All of these match findings of other earlier reports (Fageria et al., 2014; Jaramillo-Páez et al., 2017). Similarly, the binding energy peak positions of 368.01 eV (3d_5/2_) and 373.05 eV (3d_3/2_) were in tandem with what had been reported earlier as the binding energies of Ag (Figure 1I) (Pande et al., 2007; Ahmad et al., 2011).

Elemental mapping confirmed the presence of Zn and O in the ZnO nanocomposite and ZnO and Ag in the ZnO@8%Ag nanocomposites (Supplementary Figures S1A,B). This demonstrated the perfect distribution of the Ag in the different regions. Supplementary Figure S1C displays the UV-Vis absorption spectra of the nanocomposites. The peaks corresponding to ZnO absorption demonstrated a strong absorption in the UV region for the ZnO@8%Ag, with the Fermi level being established due to the transfer of free radicals from Ag to ZnO. This result further demonstrates how ZnO@8%Ag can be active in both the UV and the visible light region.

### 3.2 Photothermal and photodynamic performance

**FIGURE 2:**
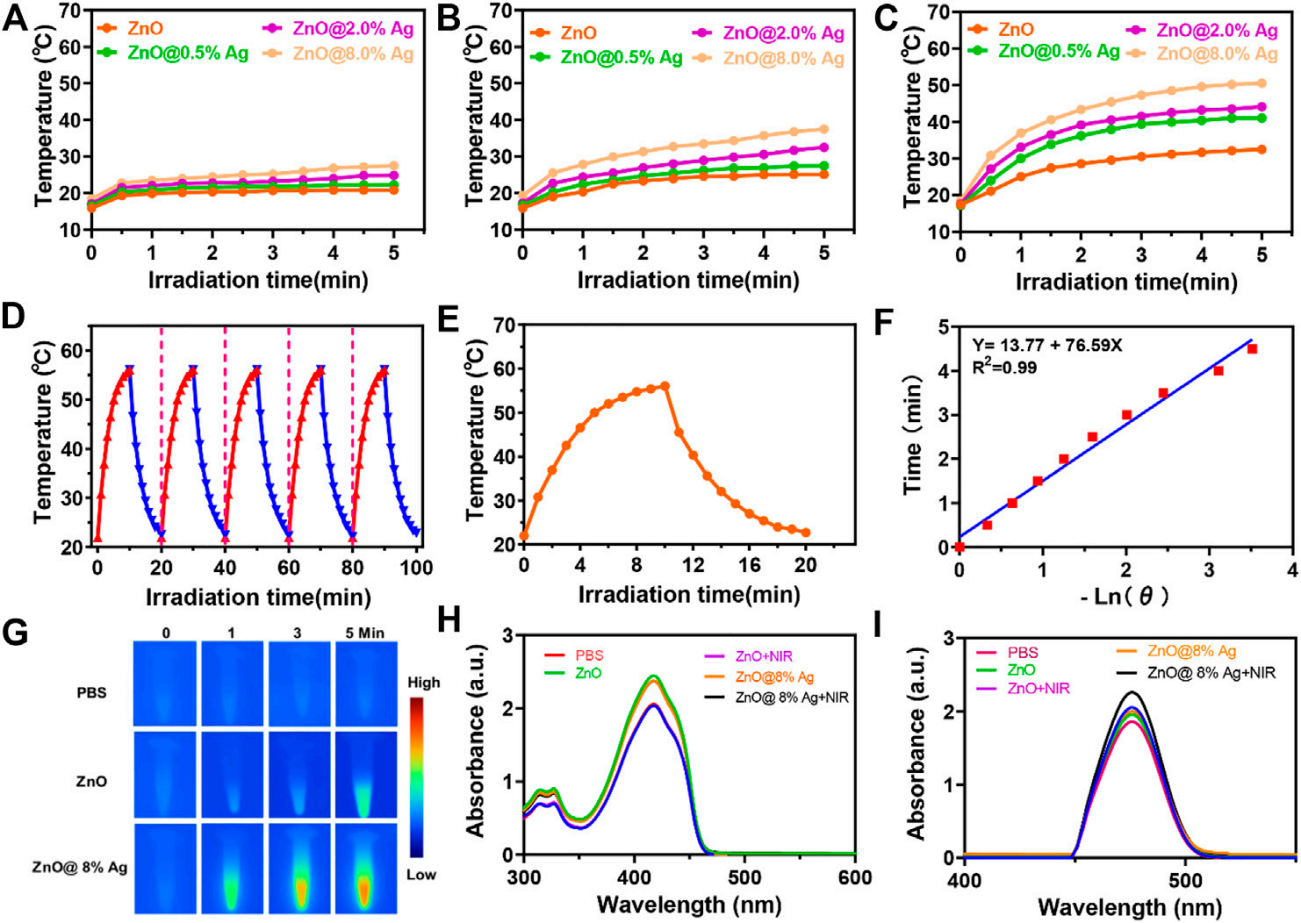
Activatable NIR photothermal and photodynamic properties of ZnO@8%Ag. **(A)** Temperature elevation curves of ZnO and ZnOAg with varying ratios of Ag (0.2%, 0.5%, and 8%) under NIR irradiation (808 nm, 0.5 W/cm^2^, 5 min), **(B)** photothermal evolution curves of ZnO and ZnO@Ag with varying ratios of Ag (0.2%, 0.5%, and 8%) under NIR irradiation (808 nm, 1 W/cm^2^, 5 min), **(C)** temperature variations of ZnO and ZnO@Ag with varying ratios of Ag (0.2%, 0.5%, and 8%) under NIR irradiation (808 nm, 2 W/cm^2^, 5 min), **(D)** recycling heating profiles of ZnO@8%Ag under irradiation for five on/off cycles, **(E)** heating and cooling of ZnO@8%Ag under NIR irradiation (808 nm, 2 W/cm^2^, 5 min), **(F)** linear correlation of the cooling time of ZnO@8%Ag, **(G)** thermal images of PBS, ZnO, and ZnO@8%Ag under NIR irradiation (808 nm, 2 W/cm^2^, 5 min),**(H)** UV spectra following the absorption of DPBF in PBS, ZnO, and ZnO@8%Ag with/without NIR irradiation (808 nm, 2 W/cm^2^, 5 min), and **(I)** spectra of changes for the absorption of OPD in PBS, ZnO, and ZnO@8%Ag with/without NIR irradiation (808 nm, 2 W/cm^2^, 5 min).

The photothermal effect was explored under 808 nm laser irradiation with power densities of 0.5, 1, and 2 W/cm^2^ for 5 min, as shown in (Figures 2A–C). Temperature higher than 50°C but lower than 60°C can destroy proteins and enzymes as well as crucial intracellular reactions (Wu et al., 2013; Pan et al., 2016; Liu et al., 2018). Upon irradiation at 808 nm, 2 W/cm^2^ for 5 min, the temperature of the solutions was elevated as follows: PBS (17–34°C), ZnO (17–32°C), ZnO@0.5%Ag (17–40°C), ZnO@2%Ag (17–44°C), and ZnO@8%Ag (17–50.5°C) at a concentration 100 μg/ml (Figure 2C). The rapid temperature elevation in ZnO@8%Ag reveals the superior photothermal property and safety that it will render to normal cells. The cooling circles (Figure 2D) and also the photothermal conversion efficiency exhibited no significant attenuation of the temperature, which reveals high photothermal stability (Figures 2E,F). In contrast, PBS, ZnO, and ZnO@2%Ag reached maximum temperatures of less than 50°C under the same conditions. Therefore, the superior photothermal performance exhibited by ZnO@8%Ag makes it promising for eradicating bacteria cells. Based on the different laser power, the enhanced temperature for an equal concentration of ZnO@8%Ag proved that the temperature rise also depended on the kind of laser power employed.

**FIGURE 3:**
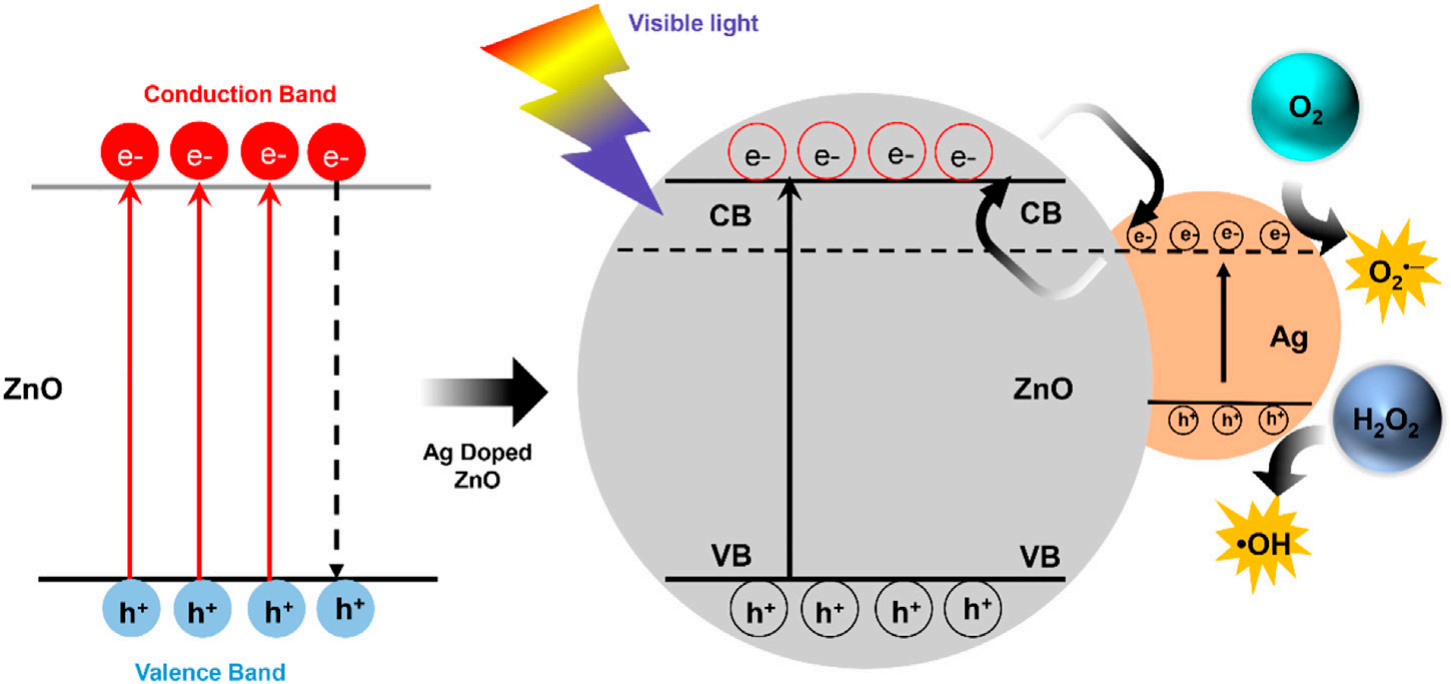
Schematic representation of the movement of electrons in ZnO@Ag under visible light.

Finally, we investigated the photodynamic property through the NIR-triggered ROS generation with DBPF and OPD. Upon exposure to single NIR (808 nm, 2 W/cm^2^) irradiation, DPBF absorption for ZnO@8%Ag gradually decreases due to the presence of ^1^O_2_ (Figure 2H), as the absorbance of the OPD increases due to the peroxidase-like activity (Figure 2I). This indicated that the Ag incorporation in ZnO increased the ROS generation of the nanocomposite. Ag in the nanocomposite acted as an efficient sink with photogenerated electrons flowing from the ZnO to Ag to prevent recombination with holes (Figure 3). Surface modification of oxides of a semiconductor influences photocatalysis, creating an electron-hole pair within the bandgap as holes are generated in the valence band and electrons in the conduction band (Hu et al., 2010; Adarakatti and Malingappa, 2016; Nagaraju et al., 2017). Photon energy that is equal to or greater than the bandgap is absorbed as an electron is promoted from the valence band to the conduction band and creating an equivalent number of holes in the valence band. However, the difference in the energy level of the conduction band of ZnO and the Fermi level of Ag presents the Ag as an efficient sink with the photogenerated electrons flowing from the ZnO to Ag to prevent recombination with the holes (Figure 3) (Nagaraju et al., 2017). These photogenerated electrons and holes can react with O_2_ and H_2_O_2_ molecules to form superoxide anion and hydroxyl radicals, respectively (Arsana et al., 2012; Kuriakose et al., 2014). This process is an indication of the role played by Ag^+^ in the enhanced generation of ROS.

### 3.3 *In vitro* antibacterial activity assay

**FIGURE 4:**
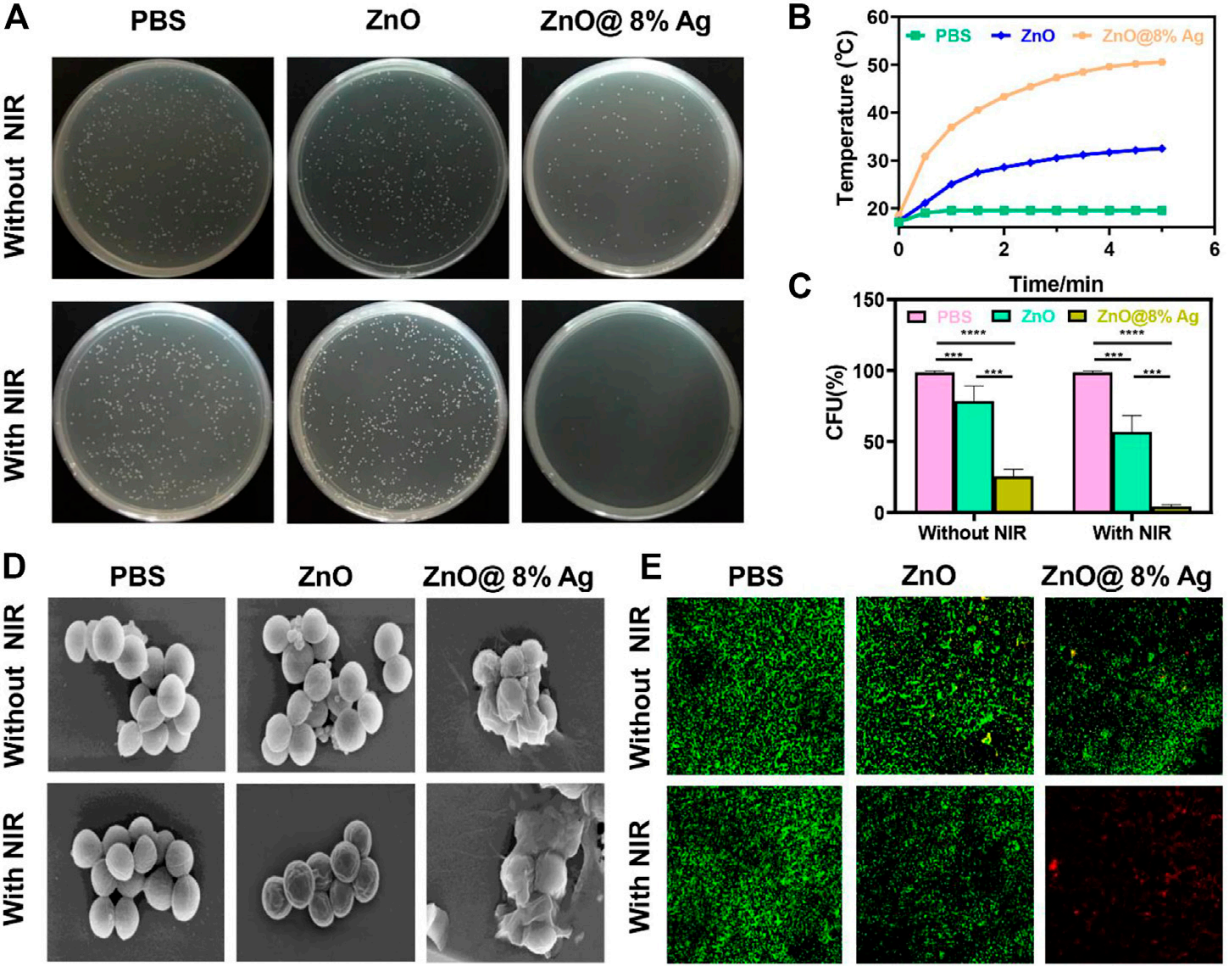
*In vitro* antibacterial activity of ZnO@8%Ag. **(A)** Bactericidal effect of PBS, ZnO, and ZnO@8%Ag against *S. aureus* with/without NIR irradiation (808 nm, 2 W/cm^2^, 10 min), **(B)** temperature elevation curve corresponding to **(A)**, **(C)** survival rate of *S. aureus* corresponding to **(B)** **p < 0.01, **(D)** SEM images of *S. aureus* treated with PBS, ZnO, and ZnO@8%Ag under NIR irradiation (808 nm, 2 W/cm^2^, 10 min), and **(E)** live/dead staining assay of *S. aureus* corresponding to **(D)**.

As shown in Figures 2C–I, ZnO@8%Ag exhibited excellent PTT and PDT ability. An Ag-based nanocomposite demonstrated effective antibacterial capacity compared to some antibiotics (Singh et al., 2017; Francis et al., 2018; Hu et al., 2018). Ag release can also be dependent on temperature, causing the photogenerated free radicals to penetrate cell walls to induce cell death**.** The material is also reported to rapidly and strongly bind to the thiol groups in enzymes and proteins located on the surface of bacteria, subsequently inducing cell death (Liu and Hurt, 2010; Shao et al., 2015). However, a higher amount of Ag can result in resistance, cytotoxicity, and aggregation of the Ag, which can further result in the instability of the nanomaterial and its ability to effectively deal with bacteria (Wiesenthal et al., 2011; Zhang et al., 2014; El Yamani et al., 2017). Nie et al. (2020) indicated that the best photocatalytic activity of Ag in ZnOAg nanocomposite was evident when the Ag content was 8%. ZnO@8%Ag (100 μg/ml) was, therefore, chosen for further future studies considering the cytotoxicity, photothermal, photodynamic, and bactericidal effects (Supplementary Figures S2, S3). Due to the aforementioned results, we evaluated the *in vitro* antibacterial effect of ZnO and ZnO@8%Ag with/without single NIR (808 nm, 2 W/cm^2^) for 10 min (Figure 4A) against *S. aureus* using the colony-forming count approach. After different treatments and cultures there was no significant reduction in the number of bacteria in the PBS, ZnO, Figure 4A. They exhibited negligible antibacterial activity with a bacterial viability rate of about 90%, although ZnO@8%Ag showed some bactericidal effect from the release of Ag^+^. Upon NIR irradiation, the colony count decreased but with a significant reduction in the ZnO@8%Ag + PTT group (Figure 4C). Results were the same for groups with/without PDT (Supplementary Figures S4A–C). These findings revealed that the release of Ag^+^ and its subsequent enhancement by the NIR irradiation proved a synergistic therapeutic effect efficient against *S. aureus*, making neither PTT nor the Ag^+^ solely responsible for killing the bacteria completely. Intriguingly, in the ZnO + PTT group, the survival rate of bacteria was substantial, indicating the negligible impact of the heat stability exhibited by ZnO, confirmed in (Figure 4B). ZnO@8%Ag exposure to a combination of PTT and PDT for 5 and 10 min against *S. aureus* (Supplementary Figures S5A,B) exhibited similar results as those observed in Supplementary Figures S3, S4A during a multimodal treatment with either PTT or PDT. To further explore the *in vitro* antibacterial effect mentioned earlier, the SEM observation and live/dead staining assay were employed. Like the control group (PBS treatment), the morphology of bacteria treated with ZnO and ZnO + PTT retained a smooth structure and remained intact. However, after treatment with ZnO@8%Ag and ZnO@8%Ag + PTT, some shrinks and wrinkles appeared on the surface of *S. aureus* (Figure 4D). The results from the live/dead staining assay confirmed these observations. Bacteria with intact membranes were stained with green fluorescence by calcein-Am, while those with damaged membranes were stained with red fluorescence by PI (Figure 4E). The intense green, evident in the PBS, ZnO, and ZnO + NIR, demonstrated that PTT alone was not sufficient to impact bacterial viability. Small red fluorescence indicated weak antibacterial activity, and strong red fluorescence in ZnO@8%Ag + PTT group indicated that the integrity of the bacteria cell membrane was greatly compromised and appeared damaged and shrunken as a result of ROS induced by the Ag^+^ released from PTT treatment. Mei et al. and others have reported that NIR promoted the effective release of Ag^+^ in killing bacteria (Tsuchido et al., 1985; Gao et al., 2018; Mei et al., 2020). Thus, the effectiveness is demonstrated by the synergistic role of Ag^+^ and PTT or PDT.

### 3.4 Biofilm inhibition test

**FIGURE 5:**
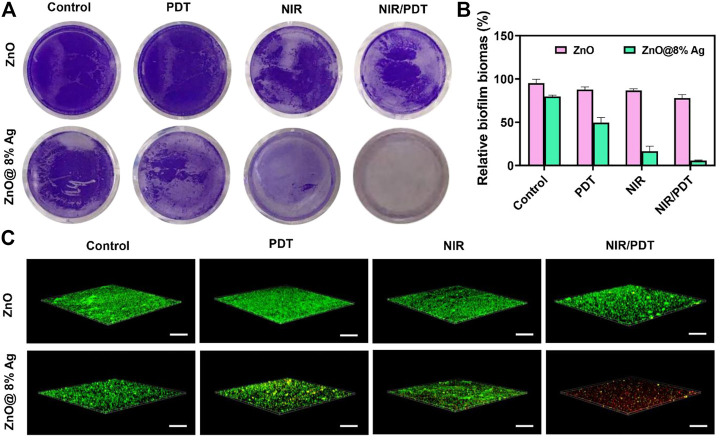
Inhibition of *S. aureus* biofilm. **(A)** Representative images of biofilm treated with ZnO, ZnO@8%Ag, ZnO + PDT, ZnO@8%Ag + PDT, ZnO + PTT, ZnO@8%Ag + PTT, ZnO + PTT + PDT, and ZnO@8%Ag + PTT + PDT, **(B)** quantitative analysis corresponding to **(A)** **p < 0.01, and **(C)** live/dead staining assay of *S. aureus* in biofilms corresponding to **(A)**.

Aggregation of sessile microbial particles and the subsequent formation of the extracellular matrix comprising a protein and polysaccharides grant microorganisms the ability to persist and survive in adverse environments (Donlan and Costerton, 2002; Vadyvaloo and Martínez, 2014; Fonseca et al., 2019). This condition makes them drug-resistant and gives them the capability to induce chronic bacterial infections. The anti-biofilm capability will validate the recognition of antibacterial materials as promising clinical translations. To study the biofilm capability, the conventional crystal violet method was employed and further confirmed with a live/dead staining assay. As shown in Figure 5A, biofilms in ZnO, ZnO + PDT group were densely distributed compared with ZnO@8%Ag and ZnO@8%Ag + PDT group. About 20 % biofilm in the ZnO@8%Ag group which was obviously due to Ag^+^ release and about 50 % in ZnO@8%Ag + PDT group which may be due to the ability of Ag^+^ under-PDT of which ZnO lacks that ability. This demonstrated the antibacterial efficacy exhibited through the release of Ag^+^. Under the biofilm treatment, in most cases, the NPs themselves are incapable of entirely eradicating the biofilm matrix and require an alternative mechanical process to deal with the biofilm matrix (Jiang et al., 2013; Fonseca et al., 2019). Compared with the ZnO + PTT group, the ZnO@8%Ag + PTT group exhibited a significantly lower biofilm rate due to the excellent photothermal ability of ZnO@8%Ag (Figure 5B). Surprisingly, all biofilms were completely eradicated in the ZnO@8%Ag + PTT + PDT group, but some significant amount of biofilm remained in the ZnO + PTT + PDT group, which indicated the effectual disruption of biofilm by the ZnO@8%Ag + PTT + PDT, a trimodal synergism. The live/dead staining assay further confirmed the antibiofilm capability of the ZnO@8%Ag + PTT + PDT (Figure 5C). Biofilm in this group was completely disrupted. In contrast, the ZnO@8%Ag + PTT group exhibited a good antibacterial effect from the PTT treatment with potential biofilm inhibition application, as the effect in the remaining groups can be considered negligible. The antibiofilm capability was performed with the same concentration used in the antibacterial test (100 μg/ml). This demonstrated that even at the low concentration, ZnO@8%Ag + PTT could effectively eradicate a significant portion of bacteria. However, the molecular mechanism of the antibiofilm activity of the ZnO@8%Ag + PTT + PDT group remains to be studied.

### 3.5 *In vitro* cytotoxicity test and hemolysis

**FIGURE 6:**
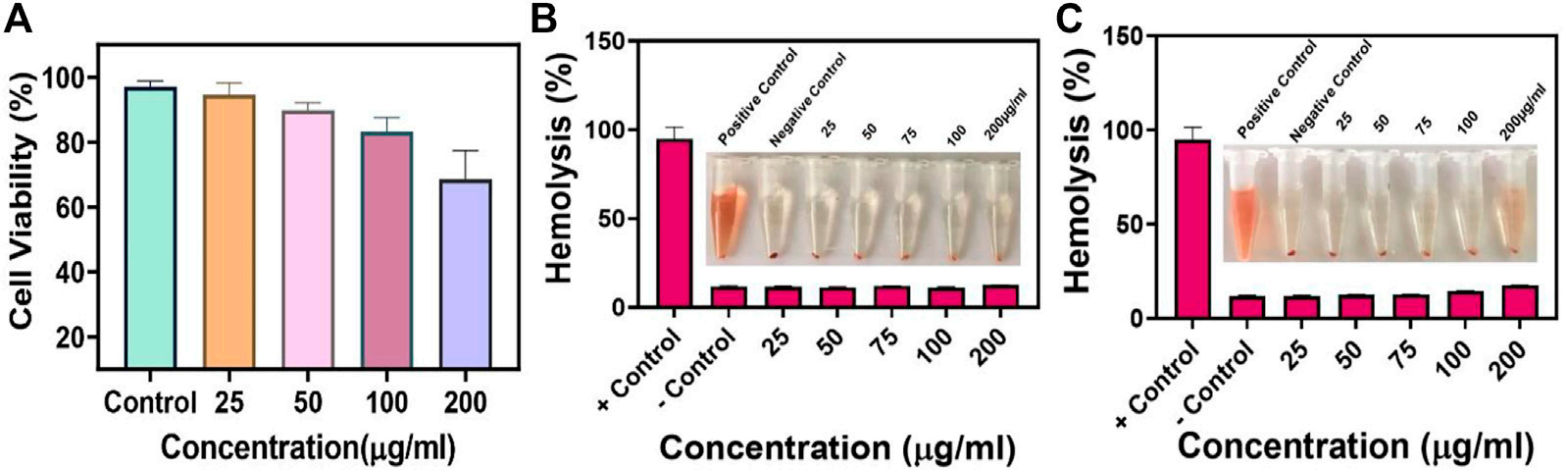
Biocompatibility of ZnO@8%Ag. **(A)** Cytotoxicity of ZnO@8%Ag under PTT/PDT in L929 cells, hemolysis rate of ZnO@8%Ag under PTT/PDT, inserted image of blood treated with varying concentrations of ZnO@8%Ag (25, 50, 75, 100, and 200 μg/ml) aqueous solutions ddH_2_O (positive control) and PBS (negative control), **(B)** after 30 min, **(C)** after 1 h.

Biocompatibility is a crucial concern for nanoparticles and a prerequisite for *in vivo* applications. Hence, biocompatibility was evaluated using the cytotoxicity test and hemolysis. L929 cells were co-cultured with varying concentrations of ZnO@8%Ag with/without PTT/PDT treatment for 5 min (25, 50, 75, 100, and 200 μg/ml) for 24 h. The cellular compatibility of ZnO@8%Ag was determined using an MTT assay. The viability of cells was over 70% even at the highest concentration of ZnO@8%Ag when exposed to PTT/PDT, indicating that ZnO@8%Ag or ZnO@8%Ag + PTT + PDT irradiation did not induce toxicity in healthy tissues. This result is shown in Figure 6A. Similarly, hemolytic evaluation of mouse erythrocytes confirmed that the ddH_2_O (positive control) group caused the release of erythrocytes from the hemoglobin, showing the induction of hemolysis. In contrast, after 30 min, ZnO@8%Ag induced negligible hemolysis with no significant difference compared to the PBS (positive control) group, even after increasing the concentration (Figure 6B). Intriguingly, ZnO@8%Ag did not induce any significant damage to the erythrocyte even after 1 h (Figure 6C), demonstrating the biosafety of ZnO@8%Ag in blood circulation and indicating good biocompatibility.

### 3.6 *In vivo* antibacterial activity and biosafety evaluation

**FIGURE 7:**
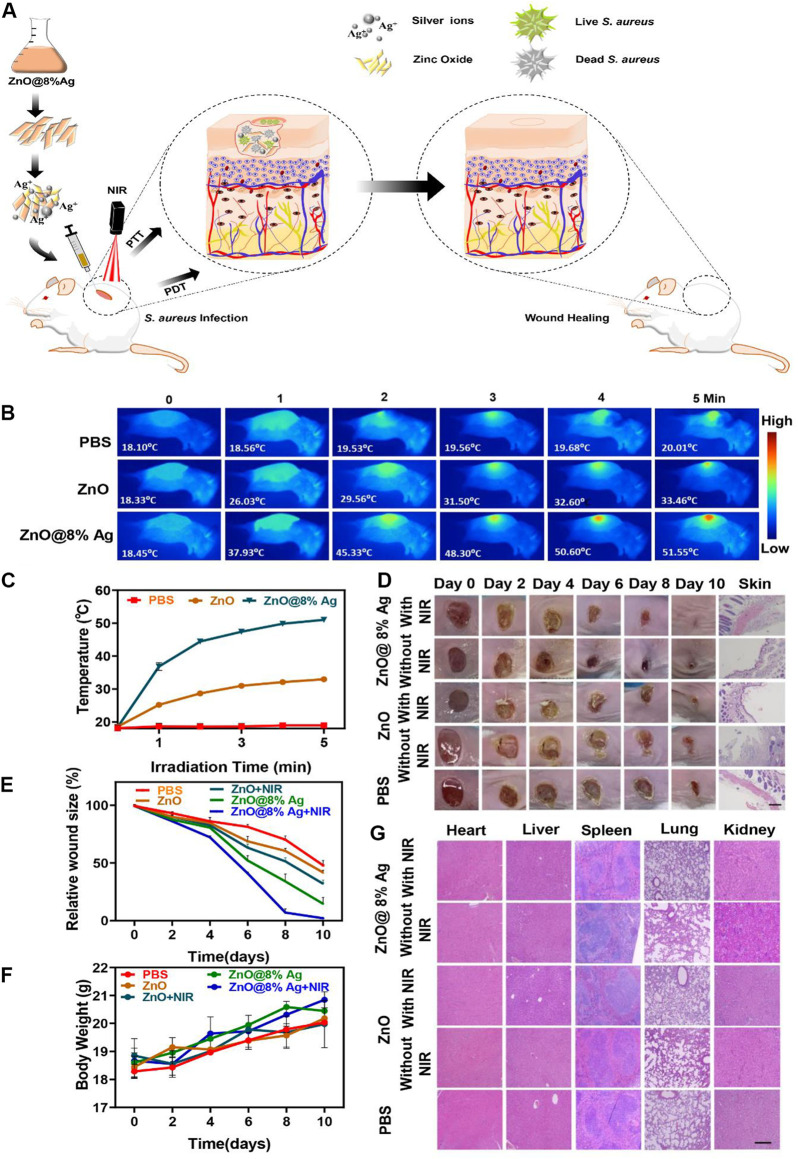
*In vivo* antibacterial activity of ZnO@8%Ag. **(A)** Schematic illustration of the fabricated ZnO@8%Ag and the corresponding antibacterial effect *in vivo* under PTT and PDT, **(B)** thermal images of PBS, ZnO, and ZnO@8%Ag under NIR irradiation (808 nm, 2 W/cm^2^, 5 min), **(C)** temperature variation curve corresponding to **(B)**, **(D)**representative images of *S. aureus* infected wound after treatment, **(E)** quantitative curve of the wound size over time for the treatment groups, **(F)** bodyweight variation curve for the different treatment groups over time, and **(G)** tissue slices of major organs (the heart, liver, spleen, lung and kidney) stained with H&E, after treatment, scale bar 100 µm.

Inspired by the *in vitro* antibacterial and biocompatibility results of ZnO@8%Ag, we further studied the *in vivo* bactericidal activity by developing an *S. aureus* infection model in BALB/c mice and evaluated the effect of ZnO@8%Ag on wound healing under NIR irradiation. The PBS and nanocomposites (ZnO, ZnO@8%Ag) were applied to the dorsal excisional (4 mm) wound inoculated with *S. aureus* (1 × 10^8^ CFU m/L, 20 µL) (Figure 7A). After 5 min of irradiation, the temperature of the wound region of the mice rose from 18°C to 52°C, without a rise in the temperature of the normal skin, compared to the PBS and ZnO groups (Figures 7B,C), demonstrating the potential of ZnO@8%Ag as a photothermal therapeutic agent. The wound healing process was monitored with digital camera, and the weight of mice within each group was recorded with a Sartorius balance. Images of the wound were taken at separate time intervals of 0, 2, 4, 6, and 10 days. As shown in Figure 7D, on day 2, all treatment groups showed scars except for the PBS group. The scar rate of the ZnO@8%Ag and ZnO@8%Ag + PTT groups was observed to be better than the other groups. On day 10, the scar in the ZnO@8%Ag + PTT group was absent, and the wound was obviously healed, demonstrating the excellent bactericidal effect of Ag from ZnO@8%Ag under NIR irradiation that enables it to facilitate wound healing by preventing wound infection (Figure 7E). In contrast, the scar remained visible in other groups with decreasing wound area in the following order: ZnO@8%Ag, ZnO + PTT, ZnO, and PBS, indicating a low bactericidal effect. Also, the weight of mice increased rapidly after day 2 of *S. aureus* infection in the ZnO@8%Ag + PTT group (Figure 7F). This may be due to the effect of the wound infection on the mice’s physiological activity and the subsequent normalization of the physiological activity by ZnO@8%Ag + PTT. However, in the control group (PBS treatment), the weight of the mice increased very slowly with time, indicating the disruption of the physiological activity, which may have led to the loss of appetite in those groups. The key organs (the heart, liver, spleen, lung, kidney and skin) sections were examined using H & E staining to evaluate the inflammatory and tissue repair response of the treatment groups under PTT treatment (Figure 7G). The results showed the number of inflammatory cells and serious infections in the control groups (PBS treatment), ZnO, and ZnO + PTT groups. The image exhibited disorganized tissue granulation with loose and irregular epithelial cells. Also, ZnO@8%Ag induced tissue regeneration, emphasizing the wound-healing ability of Ag. Remarkably, the wound treated with ZnO@8%Ag exhibited negligible inflammation, with the epithelial layer and the dermis appearing to be well organized on day 10. This phenomenon was observed in the multimodal treatment with PDT (Supplementary Figures S6A–D). This demonstrated that ZnO@8%Ag under PTT or PDT poses no long-term histological toxicity, and the material’s excellent bactericidal effect under PTT or PDT further accelerates wound healing.

## 4 Conclusion

In summary, we have successfully designed a nanocomposite (ZnO@8%Ag) with good biocompatibility and an efficient antimicrobial, antibiofilm and wound-healing effect that can combine solely with either PTT or PDT. The nanocomposite combinational use of NIR raises the temperature and unleashes ROS induction from the Ag^+^. In contrast, the trimodal treatment (ZnO@8%Ag + PTT + PDT) against *S. aureus* demonstrated a similar effect when employed multimodally, either as ZnO@8%Ag + PTT or as ZnO@8%Ag + PDT, although the PTT effect appears to be more pronounced. However, ZnO@8%Ag + PDT + PTT showed a remarkable and complete disruption of biofilm. Wound healing results further confirm the effective ability of the ZnO@8%Ag to induce negligible inflammation with accelerated tissue regeneration and wound healing under PTT or PDT. Cytotoxicity results demonstrated the biocompatibility of ZnO@8%Ag without any adverse histological toxicity to mammalian cells, even under NIR irradiation and PDT treatment. Therefore, ZnO@8%Ag under PTT/PDT presents a promising application in antibacterial biofilm and wound healing therapies.

## Data Availability

The original contributions presented in the study are included in the article and Supplementary Material. Further inquiries can be directed to the corresponding authors.
