# Supplementary material for: Multifunctional phototheranostic agent ZnO@Ag for anti-infection through photothermal/photodynamic therapy
Source: Front Chem. 2022 Nov 9;10:1054739. doi: 10.3389/fchem.2022.1054739 (PMC9682125; doi:10.3389/fchem.2022.1054739)
Supplement: Supplementary file 1 [file DataSheet1.docx]

**Multifunctional phototheranostic agent ZnO@Ag for anti-infection through photothermal/photodynamic therapy**

Enoch Obeng,^1, †^ Jiayao Feng,^2, †^ Danyan Wang,^3^ Dongyang Zheng,^3^ Bailin Xiang,^4, *^ Jianliang Shen^1,3, *^

^1^School of Ophthalmology & Optometry, School of Biomedical Engineering, Wenzhou Medical University, Wenzhou, Zhejiang, China.

^2^Ningbo Eye Hospital, Ningbo, Zhejiang, China.

^3^Wenzhou Institute, University of Chinese Academy of Sciences, Wenzhou, China.

^4^College of Chemistry and Materials Engineering, Huaihua University, Huaihua, China.

*Corresponding authors.

E-mail addresses: Bailin Xiang ( [xsz96@163.com](mailto:xsz96@163.com) ), J. Shen ([shenjl@wiucas.ac.cn](mailto:shenjl@wiucas.ac.cn)).

^†^These authors contributed equally to this work.

**Supplementary Figures**


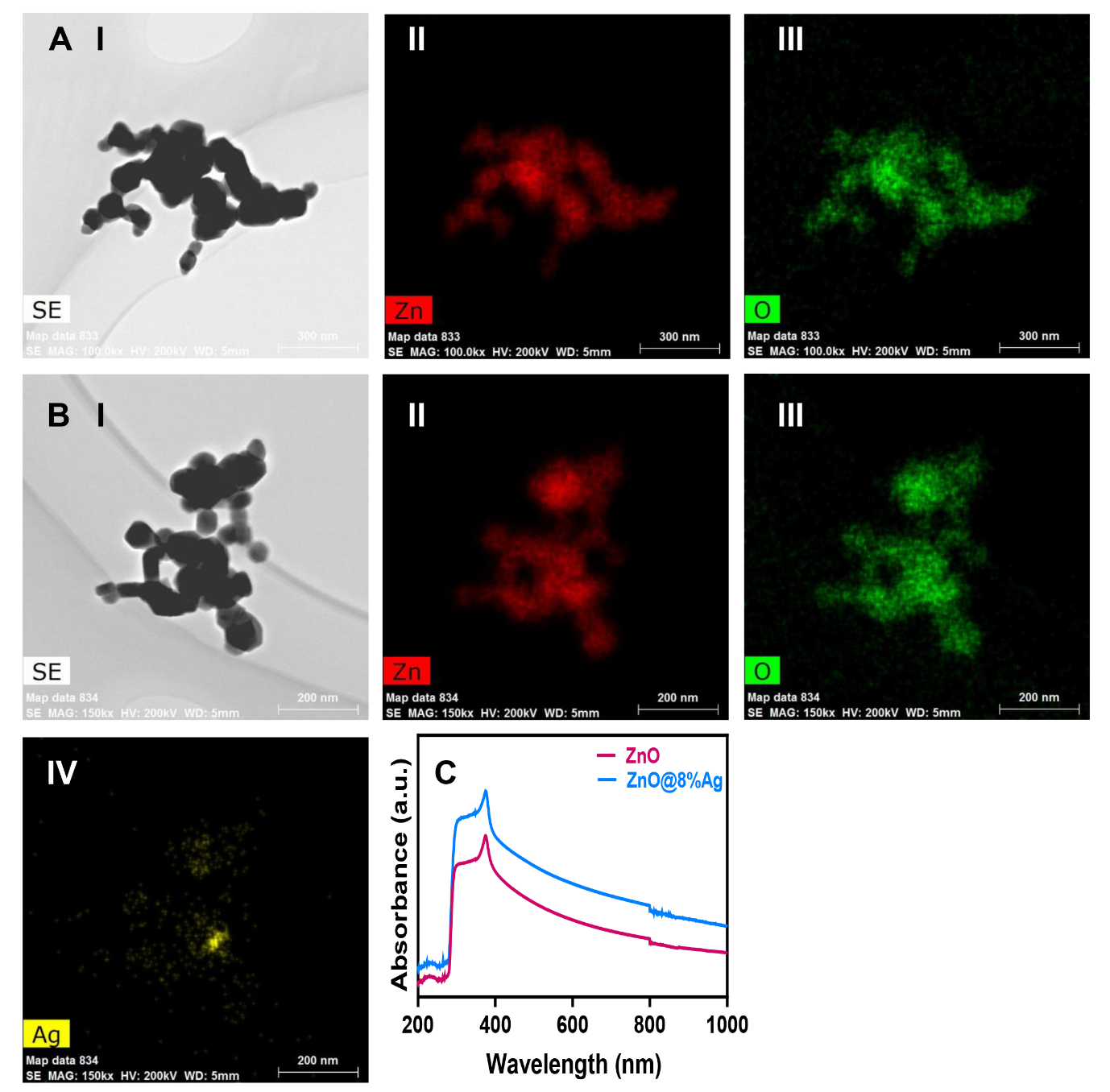


**Fig. S1** Elemental mapping (A-B) and UV-vis absorption spectra (C) of ZnO and ZnO@8%Ag.


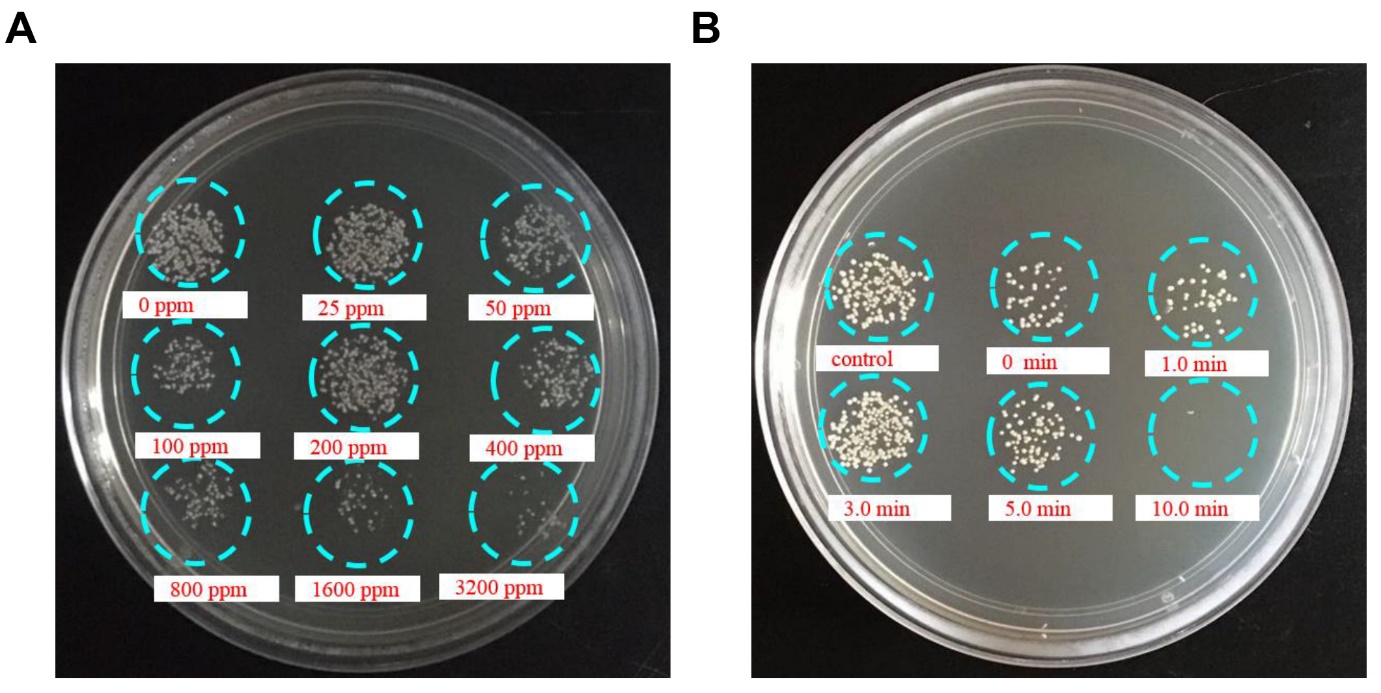
 **Fig. S2** Minimum inhibition concentration of ZnO@8%Ag in *vitro*. (A) Based on different concentrations of ZnO@8%Ag (B) ZnO@8%Ag (100 µg/mL), based on different time points.


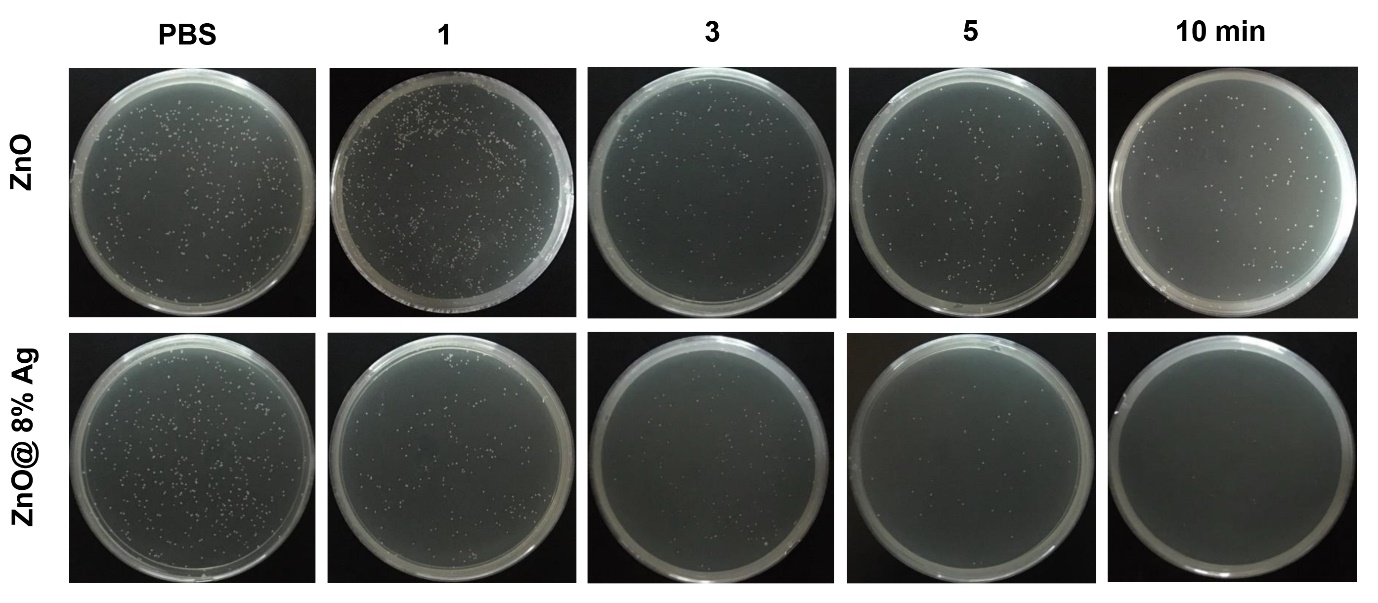


**Fig.** **S3** Representative images of *S. aureus* colonies after treatment with PBS, ZnO, and ZnO@8%Ag with/without NIR irradiation (808 nm, 2 W/cm^2^,1-10 min).


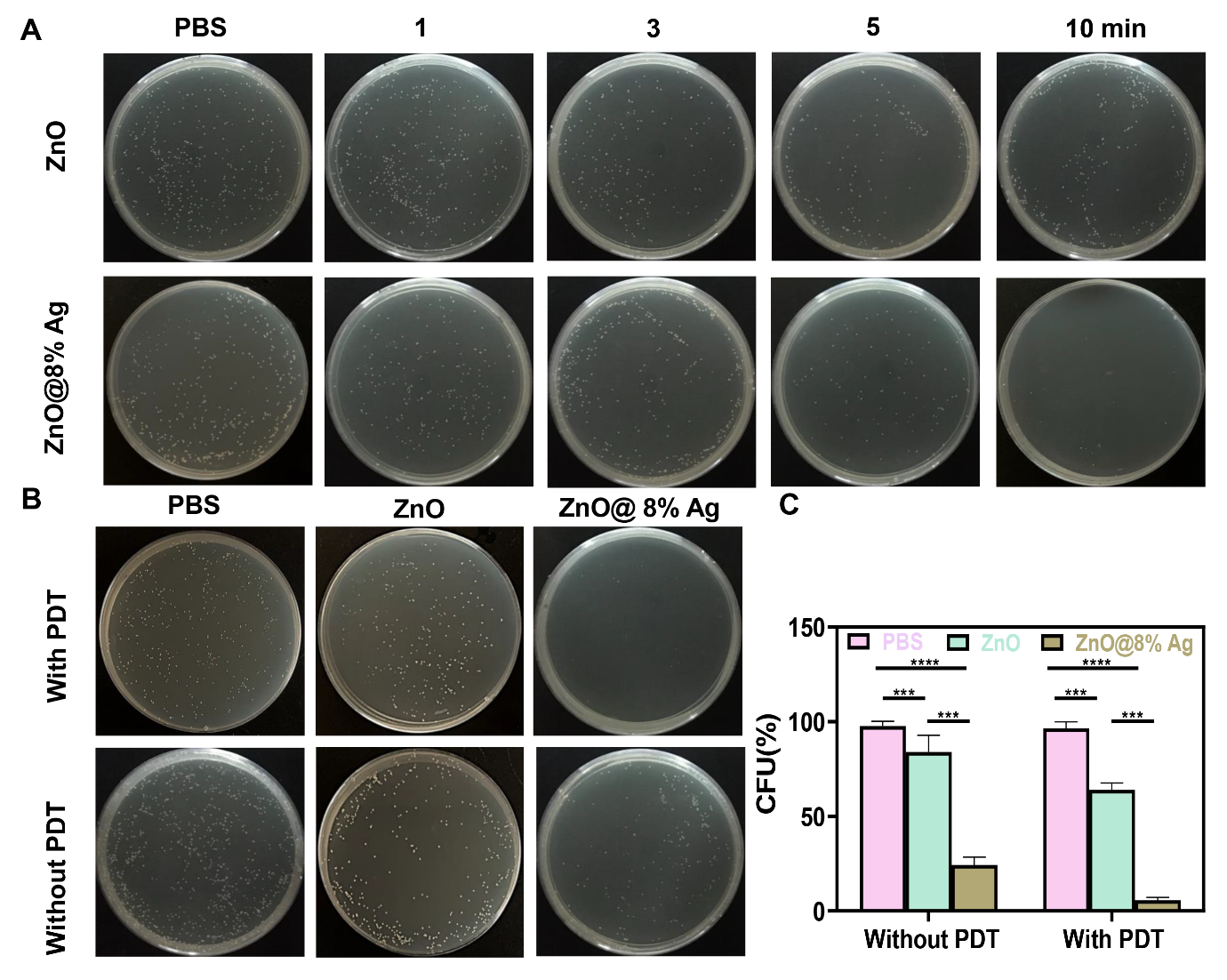


**Fig. S4** (A) Representative images of *S. aureus* colonies after treatment with PBS, ZnO, and ZnO@8%Ag with/without PDT, (B) Bactericidal effect of PBS, ZnO, and ZnO@8%Ag against *S. aureus* with/without PDT, (C) Survival rate of *S. aureus* corresponding to (B) ** p < 0.01.


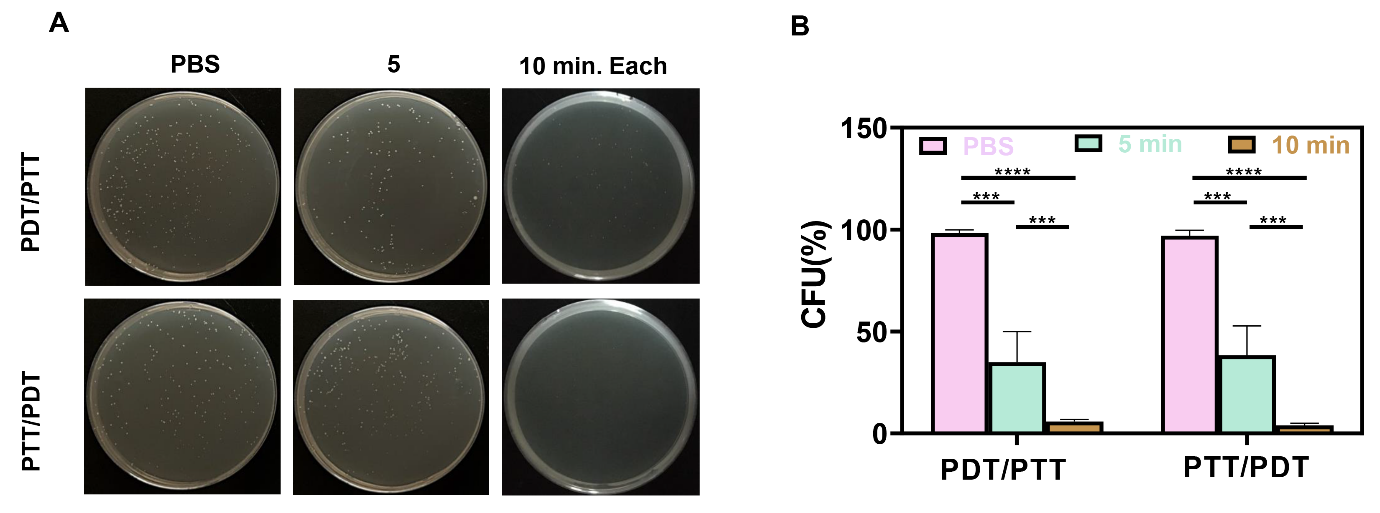


**Fig. S5** (A) Bactericidal effect of PBS and ZnO@8%Ag against *S. aureus* with/without PTT + PDT and PTT + PDT for 5min and 10min each, (B) Survival rate of *S. aureus* corresponding to (A) ** p < 0.01.


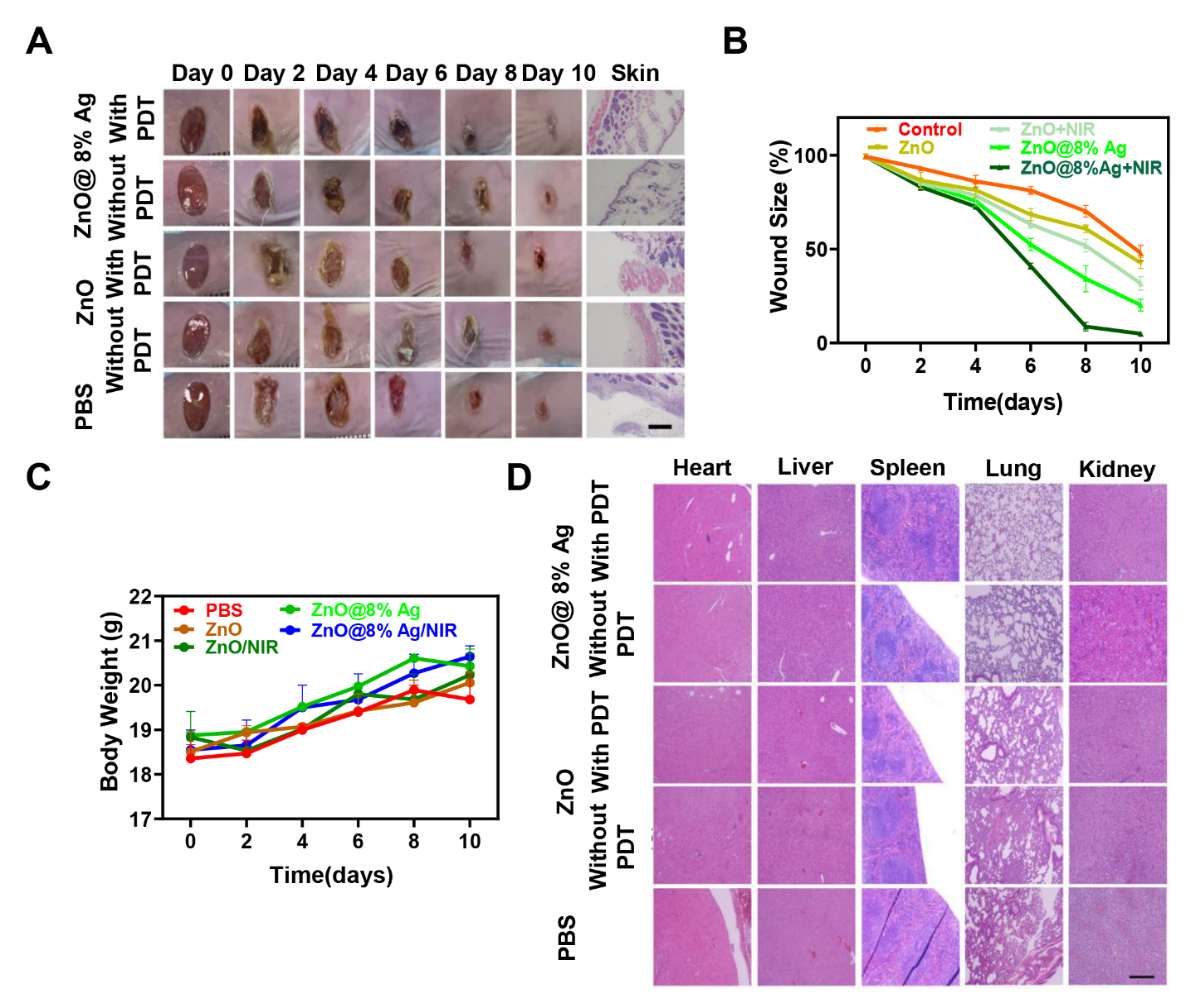


**Fig. S6** (A) Representative images of *S. aureus* infected wound after treatment (PDT), (B) Quantitative curve of the wound size concerning time for the various treatment groups, (C) Bodyweight variation curve for the different treatment groups concerning time, (D) Tissue slices of major organs (heart, liver, spleen, lung, kidney & skin) stained with H & E, after treatment, scale bar 100 µm.
